# The medical reshaping of disabled bodies as a response to stigma and a route to normality

**DOI:** 10.1136/medhum-2016-011065

**Published:** 2017-02-06

**Authors:** Janice McLaughlin

## Abstract

Disabled people are said to experience stigma because their embodied presence in the world does not fit with how others interact and use their bodies to be social participants. In response they can turn to medical procedures, such as surgery or physiotherapy, in order to reshape their bodies to more closely approximate norms of social interaction and embodiment. This paper explores how medicine plays a role in attempts to be recognised by others as normal and acceptable by minimising disability. It will do so via a focus on disabled young people, in order to explore how their emerging identities and aspirations for the future influence how they think about their bodies, what normality means and their participation in multiple activities that work on their bodies. The paper draws from an Economic and Social Research Council (ESRC) project that used a range of qualitative research methods with a group of disabled young people. The project explored ways in which participants actively worked on their bodies to be more normal and examined the disciplinary and agency dynamics involved in this work.

## Introduction

A long-term theme in medical humanities is the significance of narrative as a pathway to understand the patient's perspective. Narrative accounts produce an engagement with the meanings and values that influence how patients interact with medicine.^1^ They also allow us to understand more fully what it is to live with illness or disability and to find a way through suffering.^2^ Finally, narrative also enables us to connect the singular account to the broader cultural and social contexts that help shape that account. These contexts are important to understand, both as something that provides a richer understanding of the patient, but also as something that medical practitioners can engage with in order to better support the people they work with. The window it can provide to locating the patient in their broader social worlds can generate appreciation of what is important for them to maintain as they go through illness or live with a disabled body. It can also help us understand some of the challenges they can face because their illness or disability unsettles both their individual identity and societal norms. For example, Sparkes and Smith have used a narrative approach to explore how men respond to sports injuries that lead to permanent paralysis. They show how the men's accounts of their new lives capture the ways in which their masculinities are undermined and regained in the context of disability.^3^ It is important for men who acquire a disability to find a way to regain their masculinity because the social stigma associated with disability creates a barrier between being both disabled and masculine.^4^ Therefore, narrative research helps bring to the fore how stigma can be one of the influences on people's approach to living with illness or disability and treatments offered to them.^5^ This paper takes a narrative approach to understanding both the current stigmas associated with disability for disabled young people and how their management of that stigma influences their interactions with medicine. To do so it draws from sociological approaches to stigma, which are then used to examine the narratives of disabled young people talking about their bodies and medical interventions they have experienced.

## Sticky encounters

Sociological work on stigma is influenced by its roots in symbolic interactionism—the study of the replication of social norms and values within everyday interactions and narratives. Stigma emerges when people's ways of being in the world are counter to norms of how people are supposed to interact with each other.^6^ In classical accounts of stigma, disability is often used as an example of something that unsettles the norms of social interaction, producing what Davis^7^ famously referred to as ‘sticky encounters’. When such problems appear, disabled people attempt to avoid the social penalty of stigma by managing their embodied differences to minimise the disruption in the interactional flow.^8–10^ One of the most influential accounts of disability from this perspective is that by Goffman,^11^ in particular his argument that social expectations about how ‘normal’ bodies behave and look influence how people respond to those with impaired bodies. For Goffman, impairment is not inherently stigmatising but becomes so in interaction, when meanings are ascribed to bodies deemed to be outside the social norm.^12^ A difference becomes a stigma when others judge it to be discrediting—that is when there is a discrepancy between a person's assumed ‘virtual’ social identity and their ‘actual’ social identity.^11^, p. 12 The ‘normal’ is therefore a prized cultural status ascribed to those ‘who do not depart negatively from the particular expectations at issue’.^11^, p. 15

Influenced by a return to analyses focused on materiality and the body, there has recently been an increased interest in using stigma in research on disability and other marginalised social categories. A key theme within this work is understanding the social penalties that come with disability.^13–17^ For example, Scully builds on Davis' notion of the ‘sticky encounter’ to explore the ‘hidden labour’ disabled people undertake to negotiate the discomfort of others:

This ‘dealing with’, which entails controlling one's self-presentation, identifying what the other person needs to know or wants to feel, evaluating which strategies are needed and implementing them, producing the required responses in turn, and so on, costs significant physical and psychological energy.^18^, p. 31

This hidden labour is also captured by Garland-Thomson, who describes the work disabled people do to minimise the effects of their impairments on social interactions as a form of ‘repair’ so that the ‘fabric of the relation…. can continue’.^19^, p. 13. Acton and Hird explore the difficulties faced by those who stutter in social interactions, and argue that using a symbolic interactionist approach generates awareness of ‘examples of the microprocesses through which individuals create and maintain the differentiation between “normal” and “stigmatised”’.^20^, p. 509 Read *et al* 21 argue that disabled people may avoid the use of visible support—whether this be assistive technology or someone's help—because reliance on support can be stigmatising. Within this contemporary work, in part due to critiques of previous stigma research for being disinterested in questions of power and macro factors,^22^ there is a greater emphasis on locating specific interactions within their broader political economy. That is to question where the content of the norms that people are judged against come from and how such norms are not just the usual ways we do things. Instead socially produced values are said to reflect dominant social and cultural ideologies.^23^ Hansen *et al* provide a helpful summary of the approaches now being seen in accounts of stigma:

These theoretical frames add a much-needed structural dimension to Goffman's concept of stigma, by linking local, interpersonal strategies for managing identities and social value to larger institutional processes of the state, the exercise of power, class relations, and cultural and ideological impositions of meaning and value.^24^, p. 77

A particular theme in current stigma research is the relationship between contemporary social and political narratives about the importance of individual self-sufficiency (sometimes, perhaps problematically,^25^ placed under the heading of neoliberalism) and attributions of stigma.^26^ ^27^ Researchers argue that narratives about those who are unable to be self-sufficient, in particular those who receive welfare benefits (outside of pensions), encourage stigma. Welfare recipients are framed as individual failures—because they cannot gain employment or get well—rather than seen as experiencing particular social and material challenges (such as the absence of employment opportunities).^28^ In the context of such stigma, people feel the need to escape welfare support in order to also escape stigma and regain respectability.^29^ Stigma therefore, as Link and Phelan argue,^30^, p. 25 has ‘the capacity to impose on others a legitimised vision of the social world and the cleavages within that world’ (quoted in ref. 29, p. 1201).

The contemporary emphasis on self-sufficiency is also found in attitudes to young people trying to make the transition to adulthood.^31^ ^32^ Prout^33^ argues that contemporary young people are working through a context that requires practices of ‘self-realisation’ and which questions those apparently unable to do so. In the contexts of shrinking welfare-regimes, greater economic uncertainty and declining job security, the expectation is that young people must make their own way in the world; pursuing their own human capital in order to succeed.^34^ ^35^ For example ‘Not in Education, Employment or Training’ (NEETs), a phrase that began as a statistical classification, is now used in public and governmental narratives that emphasise that ‘NEETs’ lack character, aspiration and ambition.^36^ ^37^ An alternative account would be to highlight the social and economic structural realities that mean that young people face deeply unequal life chances.^38^ The self-realisation now asked of young people requires working on the body itself in order to succeed and be valued: ‘high modernity has produced an unprecedented “individualisation” of the body, in which meanings are privatised and the body becomes a bearer of symbolic value’ (original emphasis, ref. 39, p, 40). Individual accomplishment through care of the body is also therefore a site of interaction and stigma as some subjects are judged—both in the everyday and political rhetoric—as valued through how they care for their bodies, while others are found wanting for their comparative lack of care.

This contemporary focus on self-sufficiency and self-care are important contexts to thinking about disabled young people's approaches to medical intervention. Disability studies has a long-term critical interest in the scope of medical intervention to do more than help disabled people have a better life by improving function or alleviating pain. Instead, disability studies writers argue that there is a relationship between social stigma and medical intervention.^40^ From one perspective, medicine can be thought of as the ‘solution’ to the sticky encounter by aiding in the production of a body that is closer in look and function to normal bodies.^41^ However, from a disability studies perspective, this turn to medical solutions to respond to embodied difference is problematic.^42^ It argues that medical attempts to match social norms of how the body should be are productive in the shaping of such norms in the first place.^43–45^ Because medicine is a privileged institution in society, its participation in reshaping bodies to approximate social norms, both validates such norms as valued ways of being and also implies that one should seek to become closer to those norms. In a context where self-care and self-improvement is expected of young people, participating in medical intervention to improve function and approximate normality could be further encouraged.

The rest of the paper, drawing from empirical research, considers whether disabled young people's approach to medical intervention, both their willingness to undergo it and also what they hope to attain through it, can be linked to the wider structures informing everyday experiences of stigma. Before doing so the project's approach will be briefly summarised.

## The study

The project was an ESRC^i^ funded study of disabled young embodiments, which built on a mainly statistical study of participation and quality of life among disabled children: Study of Participation of Children with Cerebral Palsy Living in Europe (SPARCLE). The research returned to a group of young people who, as children, had been part of a small qualitative study within SPARCLE. As this group was small, we invited young people from the project who were not part of that qualitative work to join. In total, 17 disabled young people participated: 11 from the original group, 2 from the wider SPARCLE study and 4 from a local school for disabled young people. The young people recruited through SPARCLE were written to as the first point of contact. The young people recruited through the school were introduced to the project by a teacher, who provided them with information about the project (as were the others) and invited them to come forward if they were interested in taking part. The sample included 10 young men and 7 young women aged 14–20 years who lived in and around the North-East of England. Each participant had a diagnosis of cerebral palsy and all had physical impairments which affected them in varied ways. Ethics approval for the project was granted by the regional Local Research Ethics Committee of the National Health Service. As part of the project's ethical approach, participants (and parents if the participant was under 16 years) gave informed written consent at the beginning of the project, they also then gave further consent at the end of the project for the public use of the photographic work described below. The names used in the discussion below are not their real names.

The project used a range of methods that let the young people convey their thoughts as ‘capable, social actors’.^46^, p. 133 We used face-to-face narrative interviews, photography, photo-elicitation and craft-making—methods shown to be particularly effective in research with children and young people.^47^ Multimethod designs can make participation in research more interesting for children and young people by giving greater flexibility in how data are produced, and can enable different yet complementary insights into the meanings they make of their worlds.^48^ ^49^ By having participants produce images or objects that spoke to their own experiences, while holding on to more traditional narrative modes of interviewing, we aimed to allow the participants to piece together narratives that disrupted medical ways of explaining bodies and impairments. Participants could decide on the extent of their participation, and how and what they used to take part. Interestingly, despite the range of methods we employed, most opted to be part of a one-off face-to-face narrative interview only. Time was a consideration, particularly in light of the demands of school work and exams, and some preferred brevity to a more extended involvement. Seventeen took part in those interviews, with eight doing the visual work, six of whom participated in a photo-elicitation interview. Three of that six participated in the craft-making workshops. In this paper, we draw from the interviews and the photography activity. The analysis process was guided by an exploration of the narratives contained within text, image and representational artefact. We first analysed the interviews to identify patterns within the ways in which the body was spoken of. This process was done separately by members of the research team, we then worked together to compare the patterns each of us found and agreed a set of common narrative themes. The images produced then became both prompts to explore those themes further in the second interview, and also a site of analysis.

## Intervening on the body

### Stigma and passing

**Figure 1:**
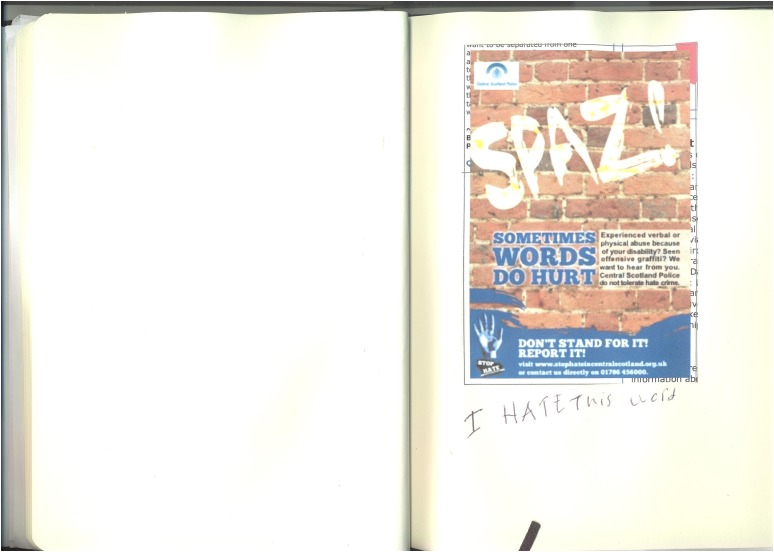
Hannah, 18, photo journal.

The young people's accounts included numerous examples of stigma triggered by how others responded to their embodied presence in the social world (figure 1). For example, Andrew (16) described mainstream secondary school as the worse place he had ever been, this was because “you have people there who don't really like people like me” (first interview). Andrew moved school due to these experiences and later also stopped going to the Scouts because people had been making fun of him and singling him out for teasing. This kind of discussion of bullying and name calling in mainstream settings such as school was common in the data. Hannah (18) captured this dynamic in figure 1 from her photo journal, alongside her own text “I hate this word”. All participants were very familiar with being stared at when moving about public space:

If you [the non-disabled interviewer] go out for a walk people don't, it is just a bit weird, people just stare at you and they think that you are not all there, when really you are all there, it is just your legs tag along with the rest of your body. (Mark, 17, first Interview)

Alongside obvious stigmatising behaviour such as bullying and staring, were more subtle ways others and ‘normal’ modes of interaction positioned participants as different and other. Examples they gave included experiences such as not being invited to friends' parties as young children, physical education teachers who did not find ways to incorporate them into sporting activities and the ways people would notice and query their use of a walking frame or walking stick. These and many other practices made the young people, from childhood onwards, aware that they were different. There were various ways they saw their differences as positive, for example, several spoke of how they thought they were more caring due to their difficult experiences. However, there were also many ways they sought to minimise their differences and spoke of wishing to be seen as normal or ordinary:

I do try to hide it, even though everyone around me says there is nothing wrong with me being disabled, yeah, but you don't get the stares and the name calling do you? (Hannah, 18, first interview)

When I was at school I always used to wear long sleeves. I always did sort of hide me, maybe my hand so that people wouldn't see, or recognise it. (Rachel, 20, first interview).

When they moved on to discuss their approach to medical intervention, it was clear that desires to be more normal and like other people influenced some of the decisions they made.

### Surgery

One of the marked commonalities across the interviews was the continued presence of medical intervention in their lives. Childhood was something they associated with hospitals, procedures and doctors. They could speak in great detail of the plates that were added and then removed; of the wires that were used to encourage muscles or tendons to stretch and bones to grow in particular ways. When not in hospital they were receiving physiotherapy at home and school, wearing splints and receiving injections of Botox or baclofen. Hannah spoke of surgery as something that had been a constant in her childhood and which had emotionally taken its toll:

I've been in and out of hospitals, I've been poked and prodded and I think I just got fed up of being like poked and prodded and operated on and I think I just got sick of being in there getting things done. (Hannah, 18, first interview)

**Figure 2:**
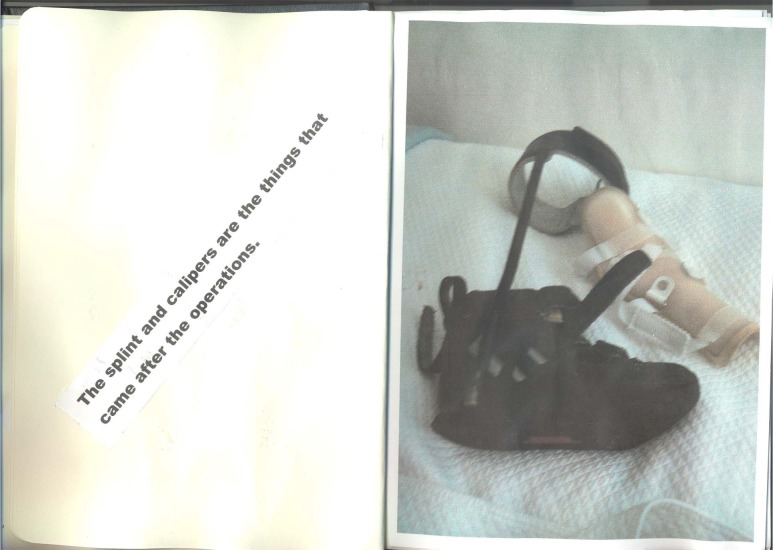
Hannah, 18, photo journal.

Hannah also captured this toll in her photo journal which displayed a number of images as shown in figure 2.

Eventually, Hannah decided in her early teens to stop having any more surgery as she did not want to endure the pain she associated with them. She also found being in hospital and interacting with doctors upsetting and disruptive to her daily life.

While the majority of participants spoke in similar ways around the pain and inconvenience of the range of medical procedures they had experienced, the majority also felt that they had benefited from them:

I think I do have a good future…. Like what I would be able to do now after I've had me operations and that. And just like I'll definitely be able to be more independent, like if I didn't have the operations. So it's making it a lot better… With me walking and like growing up. (Paul, 16, first interview)

Rachel spoke in similar ways about how she felt ‘stronger’ due to the surgeries she had, including a recent operation she had just recovered from. In both Rachel's and Paul's discussion, independence and strength were narratives they associated with their bodies being made better by surgery. They also felt that they were stronger people for everything they had been through. Medical intervention was framed as something that enabled independence both because it had helped repair the body, but also because it had changed their character. Participants often expressed the view that their experiences of medical intervention had made them stronger—mentally as well as physically.

The overarching objective the participants sought from intervention was to remain or gain greater independence as a route to normality. Independence was rarely questioned as a core symbol of a normal life. Due to this it was something they were willing to undertake further medical procedures in order to obtain.

If I hadn't had the most recent set of operations I would be in a wheelchair constantly like properly wheelchair bound, but now I can be so happy that I've done this because it is one of the best decisions I have ever made because it has given me more freedom and independence….

I can do something for myself, because after my first couple of operations I couldn't weight bear on each leg for eight weeks and bear in mind I had each leg done separately that was a like a total of about sixteen weeks that I couldn't weight bear, so for all that time, I kind of witnessed what I would have had to have done had I not had the surgery, had I been wheelchair bound. I don't like the amount of stuff that I couldn't do, just like basic things like get dressed, get washed. (Kate, 15, first interview)

Several participants had surgery not long before their involvement in our study—indeed Hannah, who spoke of stopping surgery some years before, had surgery on a leg and arm during our study. She explained that she had decided to return to surgery both to alleviate pain she was experiencing as her body grew, but also so her foot could be straightened, enabling her to walk and appear more ‘normal’. During the interview she emphasised this rationale by comparing her body with that of the non-disabled body of the interviewer:

…now it's not completely straight, they can't get it like yours, they can't get it, it's too strong but basically now it's like that, like still turned in a bit but more straighter than before. They can't get it like a normal, basically I won't get a normal foot, but it's to me it's more normal than what it was if you know what I mean by that, but it doesn't look like say your foot, but to me it's normal because it's like kind of like other people's. Does that make sense? (Hannah, 18, second interview)

Desires to look more normal and have a body more able to be independent fuelled a willingness to return to surgery, but also to work on the body on a day-to-day basis.

### Day-to-day work on the body

Alongside surgery, the majority of participants spoke of being involved in daily or regular activities geared towards keeping their bodies as fit and mobile as possible. This included physiotherapy available via healthcare, but also their own daily activities such as going to the gym, swimming, doing exercises at home, being involved in groups that included physical activity. Working on the body was a continuous thread in the young people's lives that had not lessened in significance as they got older:

I: What kind of exercises are you doing, stretching?

P: Yeah just stretching doing this [stretching ankle], going on the bottom stairs just dropping my heel that kind of thing, and just going to the wall and just pushing against the wall and just getting this leg back and then stretching here [pats right hamstring]. (Andrew, 16, first interview)

I sit and do this every night [gestures a stretching motion] to get my quad muscle working and then I will add sometimes I lie on the bed in front of the TV and with my legs stretched out to work, exercise the muscle in my groin and all this different kind of stuff, hamstring stretches that obviously I wouldn't do myself, someone would do them for me, but that kind of thing. (Kate, 15, first interview)

Associated with the exercise was a high level of self-monitoring of the body and its level of fitness: “I have to remind myself though to exercise my arm. If I forget to exercise it then it kind of ‘creeps up’” (Jamie, 18, first interview).

One important aspect of self-monitoring, more often acknowledged by the young women, was close scrutiny of weight:

I: So what do you do to manage your weight then?

P: Eat healthy. Exercise, eat fruit, go to the gym on a Wednesday.

I: What kind of stuff do you do at the gym?

P: I go on the exercise bike, the walker [treadmill], the pedal bike, and the boat [rowing machine]. (Lauren, 16, first interview)

In acknowledging gender as a factor in the importance participants gave to weight, the argument is not that the young women were simply focused on a goal of thinness as a feminine ideal. Instead, their relationship to weight was also connected to their goal of staying walking in order to be independent:

Because you can just be too skinny or you can be you know too perfect, if you know what I mean… because I need to keep my weight down. …‘cos the surgeon say obviously ‘cos my legs are weak and to keep the weight from my top half off… Yeah so I need to try and keep slim anyway, so if I get like too heavy obviously my legs are weak anyway so they'll just basically collapse or stuff… And I'll just need to use my wheelchair. So I try to keep healthy, but not too healthy, like not too slim. (Kate, 15, first interview)

Being thin was important as a marker of attractive femininity, but also if Kate was too heavy her legs would struggle to support her and she would be back in the wheelchair she had recently had surgery to avoid. However, if she became too thin, she would lose the strength to hold up her body weight and this could also place her back in the wheelchair. Ensuring she did not become too thin or too heavy required close surveillance of both what she ate and what she did in terms of physical exercise. The importance of staying out the chair, to the self-monitoring of weight by Kate and others, provides an insight into how disability generates particular dynamics of body scrutiny linked to the valuing of self-reliance and the discomfort with embodied difference.

### Stigmatising others

Final evidence of how stigma influenced participants' approaches to medical intervention is seen in judgements some made about others less involved in reshaping their bodies. The pride participants took in being able to cope with surgery and improve their bodies through daily activity and self-surveillance, also produced judgemental narratives about others they thought were not doing enough to make their body self-sufficient:

P: Erm there's a guy at my school who's got the same as me, he tends to let everybody do everything for him, and when I look at him I'm thinking you are so lazy [laughs], I'm not trying to be mean or anything but you are so lazy, I just get so sick of him. I'm thinking I've been through way worse than him, because he hasn't been through any operations or anything, or experienced that pain from what I know. If you've been through all the stuff that I've been through you'd probably be dead by now [laughs], because he probably wouldn't be able to cope [laughs]. No I'm being serious, he just annoys me.

…

I: Yeah cool, so do you think having, and I suppose it comes back to what you said about you being who you are, because of it I mean do you think it's helped you cope?

P: Yeah, more determined to be somebody. ‘Cos if I look at him I'm thinking you will go nowhere in life because you will just let everybody do everything for you and you'll let, he's just an attention seeker. (Sara, 15, first interview)

Such judgemental narratives reflect contemporary stigma around those identified as not doing enough to help themselves. Its presence in the data indicates that such stigma did not just influence the participants' approach to their own bodies. It also influenced their judgements of others. Such judgement could be made because of how much they valued independence as a core value of a normal life:

Oh it's huge it's I would say it's [under] rated sometimes I think it's absolutely massive to do things for yourself it, it gives you that sense of quite often, well carrying a bag in my eyes it's quite a good thing, it might fall off your lap three or four times but you get there in the end and yeah just things like that give you a little, little buzz. It might not be much but carrying a bag or carrying a pint back you know, carrying a drink back with having to use your hands to push it's like, obviously it's quite good and to do things yourself is a lot better than getting other people to do it for you is massive. My independence is a lot yeah. Doing things yourself is overrated, underrated sorry. (Mark, 17, first interview)

The participants' approach to surgery, their regular routines of exercise, their everyday habits of self-monitoring and their judgements of others are, at least in part, attributable to contexts of stigma, valued transitions to adulthood and societal norms of self-reliance. Their willingness to undertake surgeries and regularly participate in physiotherapy and other exercises in order to remain mobile and be independent is more than a simple choice. Such choice emerges in a context where young people experience everyday stigma for being different, and where being self-reliant and independent is valued as a normal form of life. It is understandable they are keen to be associated with those values. In their interactions with us, through their presentation of themselves as working towards a valued adult future, they presented a socially ‘credible’ person. As well as assert a particular person they were also busy working to achieve a particular body that could be associated with the credible subject. Disabled young people are not alone in trying to match their bodies to a particular social norm, however the dedication our participants enacted towards trying to do so, speaks to a context where not doing so comes with significant penalty. Our participants were conscious that adult futures were possible for them, which had not been there for previous generations of disabled young people. However, they also recognised that in working towards that adult future they should not overly rely on family to visibly support them. Nor should, or indeed could, they expect much help from welfare institutions. Instead, the credible adult thing to do was to strive towards the future in a way that was as self-reliant as possible. The participants wished for independence and an independence others would recognise as such, but knew it would not be easy. They knew they would face challenges in doing what they wanted in life due to disability and the social barriers associated with it. Their main response in our current precarious times was to concentrate on making a body as able as it could be, however temporary and hard won, to participate within the existing social order.

## Conclusion

It would be a mistake to see all the research participants' activities as a form of self-disciplining associated with stigma management; there are two important caveats to acknowledge. First, an important reason for their participation in medical intervention and regular exercise was the alleviation of significant pain and discomfort. Their growing and ageing bodies placed pressure on their impairments, seeking help for this from medicine is not inherently problematic given the benefit it can provide. Second, there is agency and pleasure in the way in which they engaged with their activities, taking pride in what they could do. Again it would be problematic to see their ‘choice’ to participate as only a product of an imposition of broader values on to them; they negotiated their way through these contexts to take value in what they could achieve.

Nevertheless, there are some social problematics evident in their regular body work. It is possible to recognise in the work they undertook what Shilling^50^, p. 74 refers to as the ‘cultural’ bodywork people do to match norms of what others will recognise as an acceptable body. What the body can do, and also the labour visible in the production of it, provide capital and credibility in the everyday battles over social position. Avoiding social stigma is one aim of the disabled young people's compliance with medical procedures. They sought a body that interacted with others in expected ways—for example, by being able to walk rather than use a wheelchair. This generates self-disciplining and self-monitoring, which can be associated with long established medical imperatives to fix the problematic body. However, as important is the social imperative associated with challenging times for all young people, where the emphasis is on self-reliance and a minimalist welfare safety net. There are productive forms of agency present in the work undertaken, in the pleasure the disabled young people took in remaking their bodies, in imagining adult futures and in their participation in choices about whether to have another surgery, in whether to swim another lap. This means that their involvement in medical intervention should not be seen as them passively going along with what medicine says. Instead, they were embedded in ongoing processes of managing and resisting stigma.

The dynamic of agency and self-disciplining involved in the participants' choices around medical intervention and working on the body, have implications for medical practitioners who work with such young people. One thing that was evident in their discussion was they were encouraged to make their own decisions about what surgery to have and what day-to-day work on the body to undertake. There is no evidence that their participation in intervention was something dictated by others. However, it is useful for medical practitioners to think about how they explore with disabled young people what lies behind their choices. To create a space for at least the acknowledgement that disabled young people undertake painful procedures to respond to the social challenges they face. It may also be useful to explore, as we have seen practitioners do, the limitations to how far medicine can take them, particularly as their bodies age and new cycles of painful intervention are required to try to sustain a ‘normal’ body.

## Footnotes

i ESRC Grant ‘Embodied Selves in Transition: Disabled Young Bodies’, the full team: Professor Janice McLaughlin, Dr Edmund-Coleman Fountain. Professor Allan Colver, and Professor Patrick Olivier.
